# Exploring legitimacy in a municipal budget decision in Switzerland: empirical insights into citizens’ perceptions

**DOI:** 10.1098/rsta.2024.0098

**Published:** 2024-11-13

**Authors:** Regula Hänggli Fricker, Thomas Wellings, Florin Zai, Joshua C. Yang, Srijoni Majumdar, Laurent Bernhard, Leopold Weil, Carina I. Hausladen, Evangelos Pournaras

**Affiliations:** ^1^ Department of Communication and Media Research (DCM), University of Fribourg, Switzerland; ^2^ ETH Zürich, Switzerland; ^3^ School of Computer Science, University of Leeds, UK; ^4^ University of Zurich, Centre for Democracy Studies, Aarau, Switzerland

**Keywords:** legitimacy, measurement, voting methods, fairness, democratic decision-making

## Abstract

This study examines legitimacy in municipal budgeting decisions, focusing on input, throughput and output dimensions. Using data from four Swiss studies, we explore how citizens assess these dimensions across traditional and innovative decision-making processes and investigate the impact of different voting methods on legitimacy perceptions. Our findings reveal that in routine processes using traditional voting, legitimacy dimensions are considered collectively. Conversely, in innovative participatory budgeting, dimensions are judged separately, involving more active evaluation. Throughput legitimacy (perceived fairness) emerges as crucial in both contexts, while input and output legitimacy's importance varies by process type. The Method of Equal Shares voting system shifts focus towards procedural fairness, increases representation and is perceived as fairer than the traditional Greedy method. However, even fair processes cannot fully compensate for outcome dissatisfaction, highlighting the complex interplay of legitimacy dimensions. This research contributes to understanding legitimacy construction in municipal decision-making, offering insights into the relationship between voting methods and legitimacy perceptions. The findings have implications for policy-makers seeking to enhance the effectiveness and acceptance of budgeting processes.

This article is part of the theme issue ‘Co-creating the future: participatory cities and digital governance’.

## 1. Introduction

Legitimacy is fundamental to the functioning of liberal democracies. It includes what Weber [1] described as a ‘belief in the existence of a legitimate order’, encompassing the perception that societal rules are binding and valid. This concept is driven by two key elements: (1) the acceptance of rules, either through agreement with their content or recognition of the authority behind them and (2) the motivation to comply with these rules based on this acceptance. The perception of legitimacy significantly influences individuals’ behaviour and plays a crucial role in maintaining the stability and effectiveness of governance and institutions. Such legitimacy perceptions impact the stability and effectiveness of governance and institutions. Legitimacy can be considered multidimensional, with literature arguing that citizens may review representative procedures, mechanisms and outcomes in their assessment of legitimacy.

In real-world politics, examining legitimacy and its multiple dimensions is particularly relevant in the context of political decision-making processes. Given the current challenges facing representative democracies, recent years have seen numerous efforts to enhance legitimacy through innovative forms of participation [2,3]. Among these democratic innovations, participatory budgeting (PB) stands out due to its widespread implementation and diverse practical applications [4,5]. PB involves citizens in budgeting decisions, typically by allocating a portion of the budget for public distribution through voting. However, citizen engagement alone does not necessarily increase legitimacy. Challenges include the possibility of certain groups mobilizing to promote their projects, potentially reducing the fairness of the process [6]. Additionally, a lack of participation from traditionally under-represented groups can also diminish legitimacy. Nevertheless, Swaner [7] argues that PB has the potential to improve government legitimacy for engaged constituents. Recent research by Yang *et al*. [8] highlights the importance of distinguishing between preference elicitation methods (how citizens vote) and ballot aggregation methods (how votes are tallied). Their findings indicate that citizens like voting formats using rankings or point systems and non-majoritarian aggregation rules (e.g. proportional representation) that enhance fairness.

In our article, we look at legitimacy of municipal budget decisions at the individual level and at two different aggregation methods. In this context, we conduct a dual enquiry, first exploring whether dimensions of legitimacy in municipal budget decisions are assessed separately or not. Specifically, we utilize indicators of input legitimacy (opportunities for participation or expressing preferences), throughput legitimacy (process quality and fairness) and output legitimacy (effectiveness of outcomes) to identify if perceptions of legitimacy are aggregated in the mind of voters. We compare a routine process (traditional budget decision with majoritarian voting) and an innovative process (PB decision with a new non-majoritarian voting method—most importantly the Method of Equal Shares). The Method of Equal Shares conceptually divides the budget equally among voters. Each voter then ‘spends’ their share on the projects they support. This proportional method differs from the traditional ‘Greedy’ method, which selects projects for funding based on popularity measured by the number of votes. While the Greedy method is straightforward, which contributes to its popularity, it may be limited in terms of its representation. The Method of Equal Shares is more proportional but also more complex and less transparent, which merits of further study. Our study aims to compare citizens’ perceptions arising from the use of these methods, shedding light on the broader implications of voting methods on perceptions of legitimacy. This is crucial, as the Greedy method remains the most widely used voting approach.

Second, we explore whether throughput legitimacy can decrease the importance of output legitimacy in municipal budget decisions. We address this topic in two different voting method settings—a majoritarian and a non-majoritarian one. The analysis was conducted using a mixture of methods, including a factor analysis, Mann-Whitney test and regression analysis. The case studies were undertaken in Switzerland, where there is a high level of trust and solid satisfaction with politics. In doing so, we can minimize potential bias associated with arbitrary or strategic voting.

This article helps to understand legitimacy in a municipal budgeting decision, as well as the link between voting method and legitimacy. To achieve legitimacy in a municipal decision-making process is highly relevant for both, policy-makers and citizens. Legitimacy has often been measured by one indicator such as the ‘belief that a political decision-making process (is) proper and just and that the decisions produced (...) ought to be accepted’ [9]; or the ‘idea of political power rightfully held and exercised’ [10]. However, there seems to be a lack of investigation of how individuals form their perceptions regarding legitimacy, what needs to be considered if the process is innovated and which indicator(s) should be used. Our article also contributes to these questions.

### (a) Research questions

— **RQ1:** What is the relationship between the various (the input, throughput and output) dimensions of legitimacy? Are they assessed separately or not and how can legitimacy be measured?

— **RQ2:** What is the role of citizens’ fairness perceptions (throughput dimension of legitimacy)? To what extent can they compensate for losing (output dimension of legitimacy)?

### (b) Legitimacy

The establishment of legitimacy is fundamental for democracies. Without this compliance, authority could only be established by implementing costly and inefficient mechanisms regulating behaviour by rewards and/or threats of punishment. Under these conditions, effective governance would be severely impaired [11,12]. Weber [1] emphasized that *beliefs* in legitimacy provide a stronger foundation for state authority compared to customs, personal interests or emotional or idealistic motivations for solidarity. Jurgen Habermas [13] further argued that the legitimacy of those who create and enforce laws in democratic systems is derived from the norms and values upheld by the citizens. Thus, citizens’ consent is key.

From a theoretical point of view, legitimacy can be considered multidimensional, with literature arguing that citizens may review representative procedures, mechanisms and outcomes in their assessment of legitimacy. We adopt such a multidimensional perspective (see table 1) on legitimacy. Scharpf [14] distinguished two dimensions—input legitimacy and output legitimacy. Input legitimacy emphasizes ‘government *by* the people’, i.e. the extent to which the individuals subject to a political decision were involved in its formulation either directly or indirectly through representatives. In other words, it refers to the opportunities of citizens to participate in political processes and the procedures introducing their preferences to the political system. Popular votes, or direct election of representatives, are examples of procedures that aim to ensure input legitimacy of political decisions. Output legitimacy focuses on ‘government *for* the people’, i.e. the extent to which the substantive policy output effectively promotes the common welfare of a community or the wellbeing of the people. The effectiveness of policies in solving collective problems or the efficiency of state agencies in delivering public services are examples of sources for output legitimacy.

**Table 1.** Overview of legitimacy dimensions.

legitimacy dimension	description
input	involvement of citizenry in the decision-making process/representative procedures
throughput	the quality of the decision-making process (e.g. its fairness)/mechanisms
output	satisfaction with the outcome (e.g. whether you win or not)/outcomes

Later, the notion of throughput legitimacy was added by Schmidt [15]. Throughput legitimacy refers to the mechanisms inside the ‘black box’ of government between input and output [15], i.e. the quality of the internal government processes of policy-making *with* the people [16]. A fair voting method, a transparent way of policy-making or a deliberative setting with discussions and decisions based on reasons are examples that can enhance throughput legitimacy.

In a similar vein, Tost [17] conceives legitimacy as a multidimensional concept consisting of judgements based on an instrumental, relational and moral dimension. Input and throughput legitimacy together can be seen as the procedural dimension of legitimacy, while output legitimacy is the instrumental dimension of legitimacy. Moreover, legitimacy consists also of political involvement (such as political interest, civic duty or subjective personal competence) or interpersonal assurance (such as interpersonal trust, personal efficacy or civic pride) (see [18]).

In our participatory process, the voting method in the form of aggregation rule is crucial [majoritarian (Greedy) versus non-majoritarian (Method of Equal Shares)]. Majority decisions tend to be in line with the preferences of a majority, while a minority is opposed to the decision outcome. Understanding the impact among the losing minority is important, as the acceptance of a voting outcome, regardless of whether the outcome went in the favour of the respective voter, underpins the functioning of liberal democracies [19]. From a perspective of output legitimacy, we would expect the majority to support the decision outcome and hence perceive the decision as legitimate. However, this suggests a lack of legitimacy for the ‘losers’ that a certain decision produces.

If a majority rule is applied, we cannot rely only on output legitimacy to create widespread wilful compliance with political decisions, as virtually any political decision is contested and creates a divide between a group that supports it and a group that opposes it. Therefore, output legitimacy alone cannot ensure legitimacy of decisions with all groups in society. As argued above, it is also far from desirable to rely on instrumental mechanisms relating to rewards and punishments, so it is a crucial function of input and throughput legitimacy to create compliance with a decision among all members of society, independently of the outcome. In this sense, the question is whether input and throughput legitimacy can partly bridge the gap between the ‘winners’ and the ‘losers’ of politics.

#### (i) Influence of the voting process (traditional versus innovative) on independence between the dimensions

Two modes of opinion formation, a passive and an active, evaluative one, can be distinguished [6]. The two modes differ in the sources of information used and the level of cognitive effort involved. In the evaluative mode, the overall judgement is created based on separate evaluations along the dimensions. This mode involves effortful attempts to form an opinion. One or the other of the three dimensions is likely to be prioritized in the judgement process. In the passive mode, individuals rely on cognitive shortcuts, or assume the legitimacy of entities, rather than engaging in active information processing. This means that citizens do not single out different dimensions. Individuals observe authorizations or endorsements from others and base their own judgements on those observations or simply accept entities that conform to their expectations rather than on their own evaluations. Particularly in ordinary, less important, or fictitious decision-making processes and phases of stability a passive mode can be expected [17,20,21]. In those situations of an overall judgement, legitimacy can be considered to be one-dimensional.

#### (ii) Role of citizens’ fairness perceptions

Marien and Kern [22] emphasize that involving citizens (fairly) is a good way to make contested decisions. However, citizen involvement alone is not sufficient to increase political support for governance. It cannot fully compensate for losing in a vote. For this, citizens also care about the outcomes of the decision-making processes. However, outcomes that have resulted through citizen involvement (particularly through forms of direct democracy) have often been considered as more legitimate, as it binds citizens to the consequences of their own decision, rather than a decision imposed by a political elite [23]. In this sense, citizens are likely to place value on both participation and outcome. There is empirical evidence to support this, with Werner and Marien [24], demonstrating that citizens place great value on output and throughput considerations. In our study, the MES (non-majoritarian voting method) can be conceived as a fairer method than the traditional Greedy one.

The remainder of this article is structured as follows. Section 2 outlines the study’s methodology by detailing the four datasets from Switzerland and the various data collection approaches. Section 3 presents the findings of the empirical analysis. It focuses on the relationships between legitimacy dimensions in different voting processes and the impact of various voting methods on citizen perceptions. Section 4 discusses the implications of the results for understanding legitimacy in municipal budget decisions. This final section also deals with the study’s limitations, suggests directions for future research and concludes with insights relevant to policy-makers and researchers interested in enhancing the legitimacy of decision-making processes in liberal democracies.

## 2. Methodology

As Sousa, Cruz and Fernandes [25] highlight, it is important to focus on local contexts when exploring indicators of legitimacy, thus avoiding the pitfalls of ‘whole-nation bias’. As such, we adopt a case study approach utilizing four datasets gathered in Switzerland. Switzerland is a country that is relatively unique in terms of its adoption of direct democracy and high levels of trust. This means it helps us to calibrate the measurement and to investigate legitimacy in a context in which the political processes work, and the decisions are accepted. It also allows us to go beyond this and see to what extent innovations are accepted. In this way, our study also serves as a benchmark for contexts in which PB is used to gain power or reinforce political dominance (e.g. [16,17]).

For our measurement of legitimacy, we rely on the multidimensional conceptualization, on indicators of legitimacy found in literature, our own thoughts and empirical pretesting, and then adapted the same indicators to all studies. Input legitimacy refers to the opportunities of citizens to participate in political processes and the procedures introducing their preferences to the political system [14]. Regarding throughput legitimacy, fairness of the process has been shown to be highly important. The literature in social psychology [11,26–28] posits that fair treatment in political and legal processes signals to individuals that they are valued and have high social status, thus increasing their self-worth and self-esteem [11]. Evoking these feelings, in turn, leads to a greater willingness to obey to authorities and view them as legitimate [11]. For the survey in Aarau, we directly formulate what fairness could mean. Output legitimacy is the willingness to accept the result or satisfaction with the outcome. You will find the exact formulations below.

For measuring latent constructs like legitimacy, one can rely on factor analysis [25]. We use factor analysis (PAF: Principal axis factoring) to explain the maximum amount of common variance using the smallest number of explanatory constructs (i.e. factors), and to analyze the (in)dependence of the factors. In other words, factor analysis allows us to analyze whether and how the variables are interconnected and understand the underlying structure or pattern.

We collected data within four local, independent, studies from different political contexts (see table 2).

**Table 2.** Overview of case studies.

	convenience sample	real-world study
traditional budgeting process (majoritarian vote, non-participatory)	Fribourg	Ehrendingen
PB process (non-majoritarian vote, participatory)	Zurich	Aarau

Two of the datasets, Ehrendingen and Aarau, utilize real-world data, whereas we rely on a convenience sample with students in Fribourg and Zurich. A convenience sample refers to a non-probability sampling that includes people who are close to hand. We selected the four cases in the following way: on the one hand, we combine traditional budgeting decisions (non-participatory, majoritarian decision in Ehrendingen and study in Fribourg) and innovative (PB) ones [PB decision with non-majoritarian voting (Method of Equal Share) in Aarau and Zurich].

**Figure 1.**
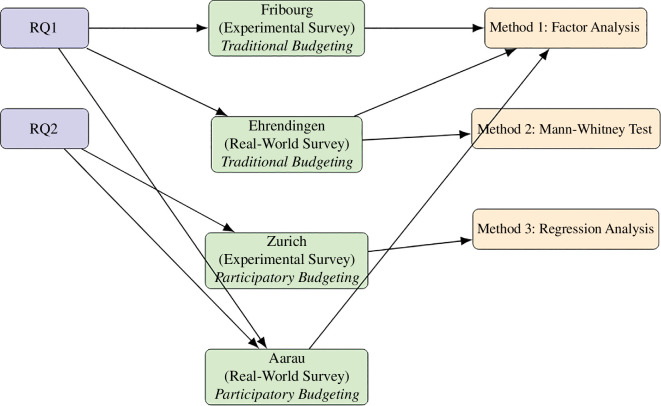
Link between research question, case study and methodology.

We expect the results (see RQ1 and RQ2) to vary depending on this dimension. On the other hand, we rely on a convenience sample (Fribourg and Zurich) and on real vote surveys (Ehrendingen and Aarau) for the traditional and the innovative budgeting process. The convenience sample in Ehrendingen was used in order to check our measurement before adopting it in the larger scale. The real vote survey then helps to overcome its disadvantage of limited sample and generalize beyond the student population. The convenience sample in Zurich helped to prepare the choice of the voting method and to deepen the insights between majoritarian and non-majoritarian voting. Furthermore, we pay particular attention to the use of the Method of Equal Shares in Aarau and Zurich. The link between research questions, case study and methodology are outlined in figure 1.

### (a) Experimental survey with students in Fribourg (vignette study)

In October 2020, we conducted a vignette study [29] at the University of Fribourg; 90 individuals completed the experimental online survey. They all assessed three distinct vignettes that were randomly distributed, resulting in a total of *n* = 270 cases. Six cases were excluded due to a high number of missing values. Initially, participants evaluated five CHF 100 000 communal projects, covering municipal finances, public spaces, parking, noise reduction and biodiversity. These projects were chosen based on a pretest and aimed to cover diverse political interests in a realistic manner. Using a slider, respondents expressed their support for each project. Subsequently, they were shown three vignettes involving municipal decisions on project allocation.

The vignettes explored agenda setting (citizen or council proposals), voting procedure [top-down (=no vote), majoritarian vote, Borda count (=a non-majoritarian vote; more in detail: A positional voting rule that gives each candidate a number of points equal to the number of candidates ranked after them. Hence, the lowest-ranked project gets 0 points, the second lowest gets 1 point and so forth. The points for each project are then totalled), quadratic voting (=a non-majoritarian vote)] and voting outcome (preferred or opposed project implemented). Preferred and opposed voting outcomes were programmed based on the previous evaluations of the projects to make the vignettes more realistic. The 2 × 4 × 2 design led to a total of 16 vignettes (refer table 3 for an overview). Participants were instructed to envision their municipality’s decision-making process and were aware of scenario variations. Legitimacy was measured as the dependent variable after each vignette using six items. We used 11-point scales reaching from 0 (*not at all*) to 10 (*completely*). Table 4 provides an overview of the wordings and corresponding legitimacy dimensions for each item.

**Table 3.** Combination of vignettes.

vignette	agenda setting	voting procedure	outcome
1	citizens	top-down	preferred project
2	citizens	top-down	opposed project
3	citizens	majoritarian vote	preferred project
4	citizens	majoritarian vote	opposed project
5	citizens	borda count	preferred project
6	citizens	borda count	opposed project
7	citizens	quadratic voting	preferred project
8	citizens	quadratic voting	opposed project
9	council	top-down	preferred project
10	council	top-down	opposed project
11	council	majoritarian vote	preferred project
12	council	majoritarian vote	opposed project
13	council	borda count	preferred project
14	council	borda count	opposed project
15	council	quadratic voting	preferred project
16	council	quadratic voting	opposed project

**Table 4.** Legitimacy items used in the vignette study.

wording	leg. dim.	mean	SD	median	max	min	*N*
I could influence the outcome of the decision.	input 1	5.17	3.11	6	10	0	264
I would feel that I had brought my opinion and interests into the decision.	input 2	5.8	3.04	6	10	0	264
I would feel the process for deciding on a project was fair.	throughput 1	5.91	2.97	7	10	0	264
I would feel that I was treated fairly in the process of deciding on a project.	throughput 2	5.85	2.87	6	10	0	264
I would be able to accept the decision well.	output 1	6.44	2.57	7	10	0	264
I would be willing to go along with the decision.	output 2	6.43	2.48	7	10	0	264

### (b) Survey of citizens in Ehrendingen

As a second study, we conducted a two-wave online survey on the annual budget vote in the municipality of Ehrendingen. The surveys ran from November to January and concerned the municipality’s budget vote for the following year. In Swiss cities, the budget must be approved by the population at the beginning of the year. In Ehrendingen, the votes took place at the end of November and used simple majority voting. In 2020, the municipal assembly (physical meeting of citizens) was replaced by a postal vote due to the pandemic. This allowed us to compare the legitimacy of different voting modalities in the field. In cooperation with the municipality, a letter with a link to the survey was sent to all residents. About 245 people with voting rights took part in the first survey (2020) and 83 in the second survey (2021), 67 of whom had already taken part in the first survey. The central question of the legitimacy of the budget decision was asked in both surveys. Six items were used to measure this. These relate to the acceptance of the decision, the perceived fairness of the decision-making process and the influence on the decision. In each case, a scale from 0 (*not at all*) to 10 (*completely*) was used. Table 5 shows the wording of these six questions.

**Table 5.** Legitimacy items used in the Ehrendingen study.

wording	survey	leg. dim.	mean	SD	median	max	min	*N*
to what extent were you able to influence the budget decision from your perspective?	2020	input 1	5.84	3.47	6	10	0	245
	2021	input 1	5.68	3.43	6	10	0	83
would you have liked to have had more influence on the budget decision?	2020	input 2	5.59	3.55	6	10	0	245
	2021	input 2	5.34	3.32	5	10	0	83
to what extent do you think the way in which the budget was voted on was fair?	2020	throughput 1	8.73	2.62	9	10	0	245
	2021	throughput 1	8.27	2.5	9	10	0	83
how fairly do you feel you were treated in the vote on the budget?	2020	throughput 2	8.67	2.69	9	10	0	245
	2021	throughput 2	8.35	2.34	6	10	0	83
how satisfied are you with the way the budget was voted on?	2020	throughput 3	8.42	2.8	9	10	0	245
	2021	throughput 3	8.02	2.59	9	10	0	83
to what extent are you willing to accept the decision on the budget?	2020	output 1	9.2	2.45	10	10	0	245
	2021	output 1	8.54	2.41	9	10	0	83

### (c) Survey of citizens in Aarau

We conducted an online survey that asked participants about a PB vote that took place in Aarau between the 12 June 2023 until the 25 June 2023. The survey consisted of two waves. The entry survey was conducted from January to March 2023 and the exit survey from July to September 2023. PB is a novel form of democratic decision-making in which community members directly participate in allocating a portion of the public budget, with the Canton of Aarau allocating 50 000 CHF to fund projects. The vote adopted cumulative voting [30] and utilized the Method of Equal Shares to calculate the voting result. The survey took place once citizens were made aware of the voting outcome. Around 3500 citizens were invited to take part in the exit survey, with 808 responding. Of which, 252 had taken part in the PB vote. As with the previous surveys, we adopted six items to measure legitimacy. These relate to the acceptance of the decision (output dimension), the perceived fairness of the decision-making process (throughput dimension) and the influence on the outcome or preference representation (input dimension). In each case, a scale from 0 (*not at all*) to 10 (*completely*) was used. Table 6 shows the wording of these six questions.

**Table 6.** Legitimacy items used in the Aarau study.

wording	leg. dim.	mean	SD	median	max	min	*N*
I was able to influence the outcome.	input 1	5.96	3.11	6	10	0	343
I feel the outcome of the Stadtidee votes accurately represents the will of Aarau citizens.	input 2	5.82	2.83	6	10	0	343
the budget should have been spent on fewer projects that are more expensive.	throughput 1	3.82	3.13	3	10	0	343
the winning projects are too similar.	throughput 2	4.31	2.73	4	10	0	343
I accept the outcome.	output 1	8.41	2.343	10	10	0	343
I am satisfied with the outcome.	output 2	6.44	2.37	7	10	0	343

### (d) Factor analyses

Three of the datasets, Fribourg, Ehrendingen and Aarau, were used for a factor analysis. This method is used to uncover underlying associations among variables, specifically focusing on how different aspects of input, throughput and output legitimacy are perceived by respondents. By identifying which elements cluster together, the factor analysis aids in revealing the latent structures within the data, indicating which factors respondents implicitly consider when evaluating legitimacy. To analyse the indicators of legitimacy in the Fribourg, Ehrendingen and Aarau studies, a principal axis factor analysis (PAF) was used. PAF is a robust method against deviations from normal distribution and different sample sizes [31]. This is crucial for our comparison of factors drawn from multiple studies, each with its own set of items, data distributions and samples. This, in combination with the focus on the common variance among variables, makes the results comparable and helps to determine the underlying factors across studies. When more than one factor was identified, we applied varimax rotation. It maximizes the variance of factor loadings within factors, which makes them more interpretable and facilitates a clearer understanding of how each dimension uniquely contributes to legitimacy. This corresponds to our assumption that the individual legitimacy dimensions could potentially be perceived as distinct constructs.

### (e) Experimental survey with students in Zurich

In our Zurich-based online experiment, we engaged 180 university students in a simulated digital PB session, pre-registered on the AEA ACT Registry^1^ and conducted online via Qualtrics. The study aimed to assess the impact of different voting input methods and aggregation strategies on voter satisfaction and perceived fairness in digital PB systems. Satisfaction and fairness ratings were both self-reported Likert scale (ordinal) questions in the survey, conducted before and after the participants were introduced to the explanations of the voting methods.

Participants first identified with a district and an urban project category within Zurich to establish a connection to the city, providing a realistic PB context. They were then presented with descriptions of 24 potential urban development initiatives, simulating real projects, and were asked to cast their votes. Following the voting phase, participants were presented with different aggregated outcomes using different voting methods. Two particularly relevant methods of aggregating votes in the context of PB were explored in our study: the Greedy method and the Method of Equal Shares.

— *Greedy Method:* This is the default voting method in most PB programs. Select projects based on vote count until the budget cap is reached, valued for its simplicity [32].
— *Method of Equal Shares:* Allocates the budget in equal shares to voters, who then fund projects of their choice, supporting proportional representation in budgeting distribution.

The survey also explored how explanations about the voting outcomes affected participants’ perceptions of fairness and trust in the voting methods. Table 7 presents summary statistics for fairness and trustworthiness ratings associated with the Greedy and Equal Shares (ES) methods, both before and after an explanation was provided to the participants in the Zurich experiment.

**Table 7.** Fairness and trustworthiness ratings before and after explanations for Greedy and ES methods.

item	method	condition	mean	SD	median	max	min	*N*
fairness	Greedy	before explanation	2.99	1.03	3.0	5	1	180
fairness	Greedy	after explanation	2.97	1.07	3.0	5	1	180
fairness	ES	before explanation	3.61	0.87	4.0	5	1	180
fairness	ES	after explanation	3.93	0.77	4.0	5	1	180
trustworthiness	Greedy	before explanation	3.07	1.03	3.0	5	1	180
trustworthiness	Greedy	after explanation	3.31	1.03	3.0	5	1	180
trustworthiness	ES	before explanation	3.44	0.85	4.0	5	1	180
trustworthiness	ES	after explanation	3.88	0.74	4.0	5	1	180

## 3. Results

### (a) One or several independent dimensions?

In the Vignette and the Ehrendingen surveys (traditional cases), we see that all items load on *one* factor (see table 8). This factor explains legitimacy quite well with a high amount of variance (between 55% and 65%). All dimensions are combined in one factor and all indicators load relevantly high on it. Citizens are in the passive mode of opinion formation [17]. In the Aarau study (innovative case), we observe a different pattern. Two independent factors are extracted, cumulatively explaining 67% of the variance. The first, and most important factor, explaining 48.5% of the variance, relies on input and output indicators. The second, less important factor, relies on throughput indicators referring to the effects of a new voting method. Since a new voting method (Method of Equal Shares) has been implemented, people consciously think about how to judge the effects of this. They are in the evaluative mode of judgement [5]. However, the second factor does not become the most important one. This indicates that input and output consideration still matter more.

**Table 8.** Factor analyses.

	vignette			Ehrendingen		Aarau	
	Vig. 1	Vig. 2	Vig. 3	2020	2021	2023	
legitimacy type	factor 1	factor 1	factor 1	factor 1	factor 1	factor 1	factor 2
input 1	0.676	0.440	0.632	0.624	0.555	0.773	−0.077
input 2	0.814	0.63	0.741	0.454	0.468	0.700	−0.205
throughput 1	0.919	0.769	0.864	0.937	0.967	−0.178	0.587
throughput 2	0.965	0.889	0.947	0.915	0.975	*−0.117*	*0.552*
throughput 3	*N/A*	*N/A*	N/A	0.945	0.957	N/A	N/A
output 1	0.670	0.796	0.836	0.832	0.752	0.597	−0.244
output 2	0.679	0.837	0.783	*N/A*	*N/A*	0.806	−0.271
eigenvalue	3.803	3.306	3.904	3.880	3.894	2.912	1.115
% of variance (V.)	63.390	55.107	65.075	64.667	64.895	48.541	18.586

### (b) Which indicators?

The factor loadings in table 8 inform us about the relative contribution that a variable makes to a factor. Perceptions of fairness in the decision-making process (*throughput dimension*) were found to load highest on the concept of legitimacy in the vignette and Ehrendingen studies. It seems the most important indicator. In Aarau, throughput indicators load on a new, separate factor. Taken all three vignette and the Ehrendingen studies together, we see that one or both indicators for *output* legitimacy are the second-best indicator for legitimacy. In the vignette study and in Ehrendingen, variables related to *input* legitimacy were comparatively a bit weaker associated with the construct, whereas in Aarau, input legitimacy was important. This makes sense. In Ehrendingen and the vignette studies, participation was limited to the casting of votes. Opportunities of citizens to participate in political decision-making processes directly or indirectly through representatives, i.e. input, was not emphasized. In Aarau, citziens’ preferences were important and represented in the vote, mirrored by the higher factor loading.

### (c) Relationship between throughput and output legitimacy in the traditional budgeting process (with Greedy voting method)

In the next step, we compared the perceived legitimacy of those who voted in favour of the vote’s outcome and those who did not vote accordingly. This analysis was conducted on the Vignette and Ehrendingen studies, which both use the Greedy voting method (tables 9 and 10). In the 2020 Ehrendingen survey, a majority of the participants (*n* = 192) voted in favour of the outcome, and a minority (*n* = 23) did not. Due to the different group sizes and a lack of variance homogeneity, we conducted a Mann-Whitney test. The vote was perceived significantly less legitimate by those who did not vote in favour of the outcome. In the 2021 survey, a majority of participants (*n* = 66) also voted in favour of the outcome, while a minority (*n* = 16) did not. Again, a Mann-Whitney test was conducted. Here, only throughput and output legitimacy were significantly perceived to be lower by those who did not vote in favour of the outcome. There were no significant differences regarding input legitimacy. Given that the vote was about accepting the budget proposed and prepared by the local executive, it makes sense that input legitimacy (influence on the budget decision) does not seem to be so relevant (as already seen above).

**Table 9.** Legitimacy differences between individuals who voted in favour of the Ehrendingen budget and those who did not vote in favour (2020).

	in favour (*n* = 192)		not in favour (*n* = 23)				
legitimacy item	Mdn	mean rank	Mdn	mean rank	*U*	*p*	*z*
input legitimacy 1	7.00	114.89	2.00	57.35	1076.50	*<*0.001	−4.287
input legitimacy 2	6.00	99.88	10.00	163.10	921.50	*<*0.001	−4.778
throughput legitimacy 1	9.00	119.74	3.00	30.83	440.00	*<*0.001	−6.650
throughput legitimacy 2	10.00	116.64	5.00	35.91	550.00	*<*0.001	−6.048
throughput legitimacy 3	10.00	116.44	5.00	41.89	687.50	*<*0.001	5.562
output legitimacy 1	11.00	120.04	5.00	36.81	583.50	*<*0.001	−6.459

**Table 10.** Legitimacy differences between individuals who voted in favour of the Ehrendingen budget and those who did not vote in favour (2021).

	in favour (*n* = 66)		not in favour (*n* = 16)				
legitimacy item	Mdn	mean rank	Mdn	mean rank	*U*	*p*	*z*
input legitimacy 1	6.00	43.56	5.50	33.00	392.00	0.109	−1.601
input legitimacy 2	5.00	39.70	7.00	48.94	409.00	0.161	−1.402
throughput legitimacy 1	9.00	45.82	7.00	25.00	272.00	0.001	−3.257
throughput legitimacy 2	9.00	45.37	7.00	26.71	301.00	0.004	−2.915
throughput legitimacy 3	9.00	46.67	6.00	23.88	253.00	<0.001	−3.512
output legitimacy 1	9.50	47.38	6.00	21.12	206.00	<0.001	−4.087

**Figure 2.**
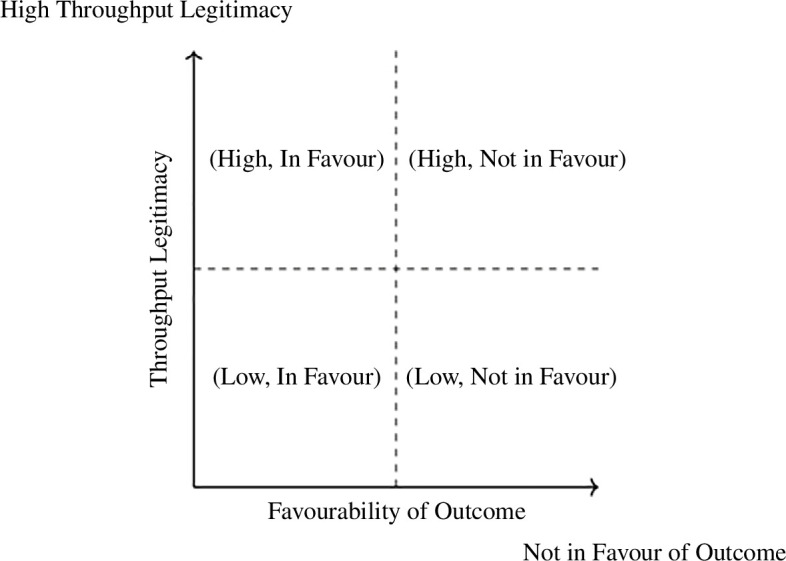
Potential groupings of respondents in terms of favourability and throughput legitimacy.

To further understand legitimacy, we conducted an analysis to explore the relationship between throughput and output legitimacy within the context of the Ehrendingen study. Specifically, we wanted to see whether perceived fairness of the process (i.e. high throughput legitimacy) can compensate for losing the vote (i.e. outcome is not in favour) and will lead to a higher acceptance of the outcome (i.e. higher output legitimacy). A visualization of the potential groupings of participants are outlined within figure 2, which shows the four potential groupings within this analysis. We used the 2020 Ehrendingen survey data. We calculated a mean score for all three throughput legitimacy variables. The internal consistency of the measure was high, with a Cronbach’s alpha of 0.95. We then created four groups based on the following two criteria: participants with low throughput legitimacy (<5.5) who did not vote in favour of the outcome (*n* = 16); participants with low throughput legitimacy who voted in favour of the outcome (*n* = 12); participants with high throughput legitimacy who did not vote in favour of the outcome (*n* = 8) and participants with high throughput legitimacy who voted in favour of the outcome (*n* = 186). Group sizes differ remarkably, due to the fact that losing the vote goes in line with a lower perceived legitimacy, as found before (see tables 10 and 11).

**Table 11.** Pairwise comparisons of output legitimacy based on level of perceived throughput legitimacy and vote.

pairwise comparison	test statistic	standard error	standardized test statistic	*p*‐value
losers dissatisfied with the process–winners dissatisfied with the process	−6.420	23.344	−0.275	0.783
losers dissatisfied with the process–losers satisfied with the process	−42.562	25.808	−1.694	0.099
losers dissatisfied with the process–winners satisfied with the process	−102.794	15.528	−6.620	<0.001
winners dissatisfied with the process–losers satisfied with the process	−36.142	27.694	−1.305	0.192
winners dissatisfied with the process–winners satisfied with the process	−96.374	18.494	−5.211	<0.001
losers satisfied with the process–winners satisfied with the process	−60.232	21.521	−2.799	0.005

Note. Pairwise comparisons of output legitimacy based on different levels of perceived throughput legitimacy (low < 5.5) and casting a vote in favour of the outcome or not were conducted using a Kruskal-Wallis test with Bonferroni correction. *p*-values less than 0.05 are considered statistically significant.

Due to the differences in the size of the groups, output legitimacy was also compared using a non-parametric procedure. Results of the Kruskal-Wallis test indicated significant differences in output legitimacy across the groups (*H* = 71.589, *df* = 3, *p <* 0.001). *Post hoc* pairwise comparisons using the Mann-Whitney *U* test with Bonferroni correction revealed specific group differences and similarities (table 11). Output legitimacy does not significantly differ for winners and losers if the process is not perceived as fair. While losing generally leads to lower and winning to higher output legitimacy, winners who perceived the process to be fairer still had a significantly higher output legitimacy (*M* = 10.03, Mdn = 11.0, SD = 1.355) than the winners who perceived it as less fair (*M* = 7.88, Mdn = 8.5, SD = 1.355). This indicates that perceiving the process as fair cannot necessarily compensate for not being in favour of the outcome but can still help to better accept the outcome when voting in favour. In line with the results above (one dimensional), both throughput and output play a role. ^2^

### (d) Relationship between throughput and output legitimacy in the participatory budgeting process (with a comparison of Greedy method and Method of Equal Shares)

In a last step, we explore the two different voting methods Greedy and Method of Equal Shares and their influence on representation and the importance of the outcome.

Table 12 compares the Greedy Method and Method of Equal Shares in Aarau. Using both voting methods to identify the percentage of representation, we see that the Method of Equal Shares can represent 58% of individuals, whereas the Greedy Method only represents 41%. The greater representation among the Method of Equal Shares sacrifices more expensive projects, which is demonstrated in the ‘Avg. Cost of Projects’ column. As is highlighted in table 6, citizens leaned towards support for not having fewer more expensive projects (as is observed with the Greed Method), with a mean of 3.82 for the question ‘The budget should have been spent on fewer projects that are more expensive’, indicating a moderate disagreement with this statement.

**Table 12.** Aarau voting representation: Greedy versus Equal Shares.

	Greedy	Equal Shares
projects selected	7	17
avg. cost of projects	7′085.71 CHF	2′905.88 CHF
budget utilized %	99.2	98.8
individual’s representation %	41.37	58.03

If we compare the means of fairness and trustworthiness between the Greedy method and Method of Equal Shares in the Zurich study (table 7), we can see that the second is higher. Thus, the Method of Equal Shares is perceived as fairer and more trustworthy.

The Ordinary Least Squares (OLS) regression analysis results from a laboratory study in Zurich, shown in table 13, explore how winning score, fairness (throughput dimension) and trustworthiness (throughput dimension) affect satisfaction (outcome dimension) across two distinct voting methods in PB: the Greedy Method and Method of Equal Shares. We chose OLS regression for its ability to robustly quantify the impact of multiple independent variables on a single continuous variable—satisfaction in this case. Satisfaction is quantified through participant responses on a scale, and the independent variables include the winning score (calculated by the budget voters win when their selected projects are funded), fairness and trustworthiness, as indicated by their survey responses.

For the Greedy Method, there is a notable positive influence of the winning score on satisfaction, with a coefficient of 1.953 and a significant *p*‐value (less than 0.05). This suggests that increased election success strongly correlates with higher satisfaction levels. Additionally, both fairness and trustworthiness are statistically significant predictors of satisfaction in this method, as indicated by their respective coefficients (0.187 and 0.351) and *p*-values. For the Method of Equal Shares, the winning score still positively impacts satisfaction but with a lower coefficient of 0.881 than with the Greedy method. Moreover, while fairness maintains a more positive relationship with satisfaction (coefficient: 0.439), trustworthiness does not emerge as a significant factor, as reflected by its higher *p-*value (0.288). This suggests that in the Method of Equal Shares, the perception of fairness is a more critical component of satisfaction than the perceived trustworthiness.

The analysis shows that the Method of Equal Shares voting method can decrease the importance of winning for satisfaction. In addition, it underscores the crucial role of perceived fairness (throughput dimension) in both voting methods.

**Table 13.** OLS regression results for satisfaction under different voting methods in Zurich, with *, ** and *** referring to significance at the levels 95%, 99% and 99.9% respectively.

coefficient	estimate	*p*‐value	estimate		*p*‐value
constant	0.561	0.023*	1.122		0.002**
winning	1.953	0.000***	0.881		0.025*
fairness	0.187	0.037*	0.439		0.000**
trustworthiness	0.351	0.000***	0.110		0.288
R-squared	0.362			0.206	
adj. R-squared	0.351			0.193	
F-statistic	33.33			15.26	
prob (F-statistic)	4.14e−17***			7.22e−09***	

At the end of the Zurich experiment, participants were asked a critical incentivized question: ‘Do you prefer the winning projects to be selected based on Method A (Greedy) or Method B (Method of Equal Shares)?’ The voting method selected by the collective decision of the participants would directly influence their pay-off. Our objective was to determine which type of legitimacy—throughput legitimacy (encompassing fairness, trust and level of understanding of the process) or output legitimacy (satisfaction)—most influences participants’ final choice between the two voting methods. Since voting methods affect solely to the calculation process and the outcome, they are distinct from how voters cast their votes. Hence, our analysis focuses on throughput and output legitimacy.

To explore participants' preferences regarding these voting methods, we conducted a *logistic regression analysis* (table 14), chosen for its effectiveness in predicting binary outcomes. The dependent variable in this analysis is the participant’s voting method preference. The independent variables are the differences in satisfaction, fairness and trustworthiness scores between the two methods, derived from survey responses.

**Table 14.** Logistic regression results predicting incentivized choice.

predictor	coefficient	SD	*z*-value	p > |*z*|
constant	0.634	0.251	2.531	0.011
satisfaction diff	0.9300	0.190	4.885	<0.001
fairness diff	0.548	0.247	2.217	0.027
trustworthiness diff	0.362	0.311	1.160	0.246
understanding diff	0.456	0.248	1.838	0.066

The difference in satisfaction (*satisfaction_diff*) emerges as a highly significant factor, pointing towards a strong influence of output legitimacy on participants’ preferences on voting methods. While fairness difference (*fairness_diff*) also plays a notable role in influencing choice, trustworthiness and understanding differences do not reach statistical significance, suggesting their impact is comparatively less decisive in this context. Thus, the analysis indicates that participants’ decisions are guided most significantly by their satisfaction with the outcomes, followed by some degree of fairness perception, underscoring the relatively important role of output legitimacy.

## 4. Discussion and conclusions

This study contributes to the ongoing debate in the scholarly literature about the nature and measurement of legitimacy in democratic decision-making processes. Conducted in Switzerland, a country known for its strong tradition of direct democracy and high levels of political trust, the research provides insights into how citizens perceive legitimacy in both traditional and innovative budgeting processes.

This empirical contribution found that the perception of legitimacy dimensions (input, throughput and output) varies depending on the type of decision-making process (RQ1). In traditional budget decisions with majoritarian voting, citizens consider legitimacy overall, while in innovative PB processes with non-majoritarian voting methods, individual dimensions are assessed. This suggests that innovative processes evoke a more conscious perception, in our case of procedural fairness. In traditional decisions, outcomes and processes (throughput) serve as important indicators for overall judgement, while input is less important.

Our analysis also revealed that the Method of Equal Shares voting system significantly shifts citizens' focus towards procedural fairness (throughput dimension), compared to the traditional emphasis on winning (outcome dimension) in the Greedy method. This new voting method *was found to increase representation and* was perceived as fairer and more trustworthy by participants. However, the study also demonstrated that even a seemingly fair process cannot fully compensate for dissatisfaction with the outcome (RQ2), thus highlighting the complex relationship between the various dimensions of legitimacy.

The findings of this empirical analysis highlight the importance of context in legitimacy perceptions, a factor often overlooked in previous research. It demonstrates that novel voting methods, such as the Method of Equal Shares, can shift citizens' focus towards procedural fairness and bring citizens to the evaluative mode of information processing, and potentially enhancing overall legitimacy. This finding is of particular importance worldwide, given that many democracies are exploring ways to increase citizen participation, trust in governance and ultimately legitimacy. However, this contribution also reaffirms the complexity of legitimacy, showing that procedural fairness alone cannot fully compensate for unfavourable outcomes, thus contributing to the ongoing scholarly discussion about the relative importance of process versus results in democratic legitimacy.

It is important to recognize that these findings, while contributing to the understanding of legitimacy perceptions, are subject to certain limitations. The results are comprehensive within the Swiss context and can be expected to apply to other liberal democratic system that allow participatory decision-making. However, our study invites further research across different political systems. This should also extend to less established democracies and non-democratic regimes, where output legitimacy emerges as crucial for policy success [33]. In such a context, the link between a decision and the support for the government more generally (link between micro- and macro-level) also becomes important.

While our use of convenience samples and student participants in some studies limits the generalizability of the findings, the robustness of our analytical methods, such as PAF and OLS regression, ensures that the findings are comparable. The student samples provided important initial explorations and a solid foundation for the following studies with more diverse and larger samples. We see that the real-world sample does not change the findings. In the Ehrendingen study, we employed non-parametric procedures to appropriately handle the small sample sizes. Additionally, the field studies’ real-world context mitigates the lower external validity of the experimental studies using hypothetical voting scenarios. Furthermore, we acknowledge that our quantitative approach may miss nuances associated with individual citizens perceptions of legitimacy. Therefore, we would encourage a qualitative follow-up study to offer a greater understanding of the results presented within this article. Nevertheless, the findings from this article have important implications, particularly for policy-makers interested in maximizing the legitimacy of the decision-making process.

Our claim that these findings could apply to other liberal democratic systems is based on three factors. First, shared democratic principles may have the consequence that liberal democratic systems generally share core principles such as citizen participation, rule of law and government accountability. These commonalities suggest that citizens in different liberal democracies might evaluate legitimacy using similar criteria. Second, there is a trend towards more participation to overcome losses in terms of legitimacy. Indeed, many liberal democracies are exploring or implementing PB and other innovative decision-making processes. This shared trend indicates that the findings about how citizens perceive legitimacy in these new contexts could be observed well beyond Switzerland. Third, the conceptualization of legitimacy (input, throughput and output) is based on widely accepted theoretical frameworks in political theory, which are certainly not unique to the Swiss context.

However, it is crucial to acknowledge that political cultures, institutional arrangements and historical contexts vary significantly across liberal democracies. Factors such as levels of trust in government, existing participatory traditions and socio-economic conditions could influence how these findings translate to other contexts. Therefore, while the results provide valuable insights that could be applicable to other liberal democratic systems, further comparative research would be necessary to confirm and refine these expectations across different national and cultural contexts. More specifically, Switzerland can be considered a ‘least likely’ case: Given Switzerland’s high levels of trust and satisfaction with politics, it could be considered a ‘least likely’ case for observing changes in legitimacy perceptions. If changes are observed here, they might be even more pronounced elsewhere.

## Data Availability

Experimental survey with students in Fribourg (vignette study). The data from the experimental survey with students in Fribourg have been published on the data repository Harvard Dataverse as a replication set for a paper submitted to the Swiss Political Science review [34]. Survey of citizens in Ehrendingen data file is attached (will be put on a permanent repository later this year). Survey of citizens in Aarau data file is attached (will be put on a permanent repository later this year). Experimental survey with students in Zurich. Please find information here [35]. Supplementary material is available online [36].

## Footnotes

^1^

https://www.socialscienceregistry.org/trials/11021

^2^
The analysis could not be applied to the 2021 survey as there were too few respondents in the respective groups at this level of detail.
